# Supplementary material for: High-Resolution Cryogenic Spectroscopy of Single Molecules in Nanoprinted Crystals
Source: ACS Nano. 2024 Jul 16;18(33):21886–93. doi: 10.1021/acsnano.4c02003 (PMC11342377; doi:10.1021/acsnano.4c02003)
Supplement: Supplementary file 1 — nn4c02003_si_001.pdf [file nn4c02003_si_001.pdf]

High-resolution cryogenic spectroscopy of single molecules in nanoprinted crystals

Mohammad Musavinezhad,[1,2] Jan Renger,[1] Johannes Zirkelbach,[3] Tobias Utikal,[1] Claudio U. Hail,[4] Thomas Basché,[5] Dimos Poulikakos,[6] Stephan Götzinger,[1,2,7] and Vahid Sandoghdar[1,2]

[1]*Max Planck Institute for the Science of Light, D-91058 Erlangen, Germany*

[2]*Department of Physics, Friedrich Alexander University Erlangen-Nuremberg, D-91058 Erlangen, Germany*

[3]*Faculty of Physics, Ludwig-Maximilians–Universität München, D-85748 Garching, Germany.*

[4]*California Institute of Technology, Pasadena, California 91125, USA*

[5]*Department of Chemistry, Johannes Gutenberg-University, Mainz 55099, Germany*

[6]*Institute for Mechanical Systems, ETH, Zurich, Switzerland*

[7]*Graduate School in Advanced Optical Technologies (SAOT), Friedrich Alexander University Erlangen-Nuremberg, D-91052 Erlangen, Germany*

I. RESONANCE LINEWIDTH FITTING

In single molecule studies, the source of spectral fluctuations is usually modeled as a molecule interacting with one or many two-level systems (TLSs) in the host matrix[1, 2]. Depending on the characteristic timescales τ_c and the amplitude of spectral fluctuations caused by the TLS dynamics, the shape and width of molecular resonances can deviate from a lifetime limited Lorentzian line shape.

In this context, the fast spectral fluctuations ($\tau_c <$ excited state lifetime) are "motionally narrowed", resulting in Lorentzian shapes[1, 2]. However, slower TLS dynamics that result in fluctuations faster than the measurement time can lead to line broadening and the resonance is best described by a Voigt function. Figure S1a shows one example where the laser frequency is scanned over the 00ZPL of a molecule for about 100 ms. Given the measurement uncertainty, which is mainly limited by shot noise, both Lorentzian and Voigt models lead to a reasonable description of the data. In this case, using a Lorentzian model for fitting single frequency scans is preferred because of its lower number of free parameters and less computational complexity.

In Figure S1b, the measurement time is artificially increased by averaging the photon counts over 180 repeated frequency scans over the same molecule. In this case, the spectral diffusion rate becomes faster than the measurement time and the deviation from a Lorentzian model is apparent.

Finally, we consider the spectral fluctuation that are at the timescales close to the measurement time. In such cases, the resonance can undergo a resolvable frequency shift along with the sampling of the 00ZPL. As a result, the line shape can deviate from both models and fitting the data is not justified. To avoid this issue, we scanned over each molecule for at least 10 times and rejected the scans that were not Lorentzian. In Figure 3c of the main manuscript, only the molecules that were successfully fitted by a Lorentzian for at least 5 times are reported.

II. DIPOLE ORIENTATION STATISTICS

We analyzed the orientation of the transition dipole moments to investigate the alignment of DBT molecules with respect to each other. For this analysis, each molecule is denoted by M_{ik}. Here, i is the index of the molecule and k indicates the host nanocrystal (NC). The transition dipole orientation ϕ_{ik} of molecule M_{ik} is considered as a random variable Φ following the probability density function

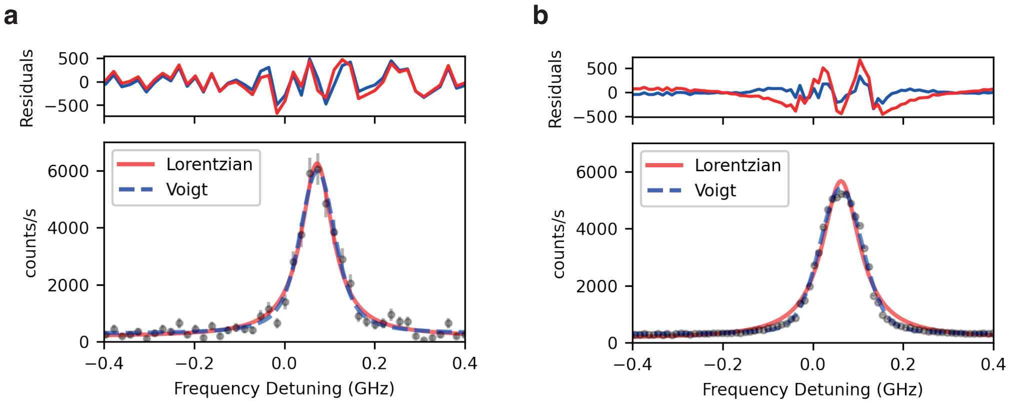

FIG. S1. Resonance linewidth fitting. (a) Excitation spectrum of a molecule in a single frequency scan. Curves show best fits to a Lorentzian and a Voigt function. Fit residuals shown on top. (b) Average detected photon counts as a function of laser detuning in 180 repeated measurements. Curves show best fits to a Lorentzian and a Voigt function. Fit residuals shown on top.

(PDF) $P_\Phi(\phi)$. We model this function as

$$P_\Phi(\phi) = a\mathcal{N}(\phi; 0, \sigma_0) + \frac{1-a}{2}\mathcal{N}(\phi; \theta, \sigma_1) + \frac{1-a}{2}\mathcal{N}(\phi; -\theta, \sigma_1), \tag{1}$$
$$\text{with} \quad \mathcal{N}(\phi; \theta, \sigma) = \frac{\exp(-(\phi-\theta)^2/2\sigma^2)}{\sigma\sqrt{2\pi}},$$

where $P_\Phi(\phi)$ is assumed to be independent of the crystal index k and the measured values ϕ_{ik} are statistically independent. Here, the angles are measured with respect to the main alignment axis, corresponding to the first term in Eq. (1). To estimate the free parameters $(\sigma_0, \sigma_1, \theta, a)$ in Eq. (1), we analyzed the statistical profile of orientation differences between molecules. We defined

$$\Delta\phi_{ij}^{(k)} = \phi_{ik} - \phi_{jk} + m\pi \qquad \text{with} \quad m \in \mathbb{Z}, \tag{2}$$

the difference between the dipole orientations of M_{ik} and M_{jk}. Without loss of generality, m is chosen such that $\Delta\phi_{ij}^{(k)} = -\Delta\phi_{ji}^{(k)} \in (-\pi/2, \pi/2]$. The resulting PDF for $\Delta\phi_{ij}^{(k)}$ can be written as the convolution of $P_\Phi(\phi)$ such that

$$\begin{aligned} P_{\Delta\Phi}(\Delta\phi) =& P_\Phi(\phi) * P_\Phi(-\phi) \\ =& a^2\mathcal{N}(\Delta\phi; 0, \sqrt{2}\sigma_0) + \frac{(1-a)^2}{2}\mathcal{N}(\Delta\phi; 0, \sqrt{2}\sigma_1) \\ &+ a(1-a)\mathcal{N}(\Delta\phi; \pm\theta, \sqrt{\sigma_0^2+\sigma_1^2}) \\ &+ \frac{1}{4}(1-a)^2\mathcal{N}(\Delta\phi; \pm 2\theta, \sqrt{2}\sigma_1). \end{aligned} \tag{3}$$

The dashed line in Figure 4d is a fit using Eq. (3). The main advantage of fitting $P_{\Delta\Phi}(\Delta\phi)$ instead of $P_\Phi(\phi)$ is that it circumvents the additional uncertainties in alignment of the main axis for different NCs.

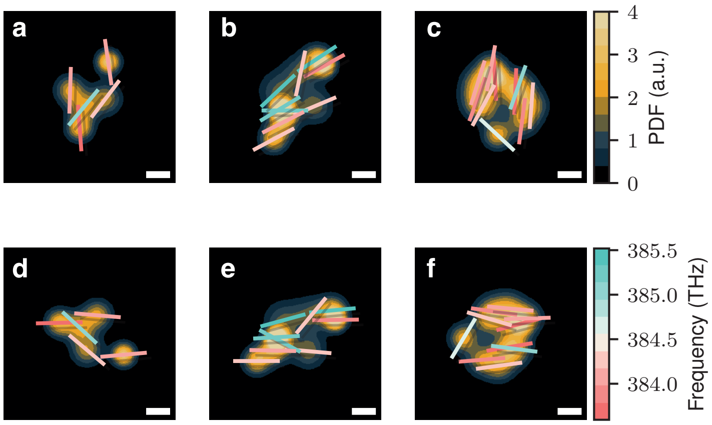

FIG. S2. Aligning emitters orientation for crystal size estimation. Scale bars show 200 nm. Color bars are the same for all panels. (a-c) Three nanocrystals are presented with the measured location $\mathbf{x}_{ik}$ and dipole orientation ϕ_{ik} of embedded DBT molecules. The most common orientation of each NC Φ_k is calculated. (d-f) The data from (a-c) are aligned through a rotation around the center of mass of molecules location. The majority of dipoles are along the horizontal axis.

III. OPTICAL CHARACTERIZATION OF THE CRYSTAL SIZE

To characterize the lateral size of printed Ac NCs, we analyzed the spatial distribution of 261 molecules within 61 NCs in Figure 5e. For each molecule M_{ik}, the transition dipole orientation ϕ_{ik} and the location $\mathbf{x}_{ik}$ is measured. Three examples of the measured values are presented in Figure S2a-c. For molecule M_{ik}, we generate a normalized Gaussian spot $G_{ik}(\mathbf{x})$ with standard deviations equal to the estimated localization accuracy as PDF of its location $\mathbf{x}_{ik}$, i.e. $G_{ik}(\mathbf{x}) = \mathcal{N}(\mathbf{x}; \mathbf{x}_{ik}, \sigma_{ik})$. The underlying images in Figure S2a-c represent the sum of these PDFs for individual NCs, i.e. $\sum_i G_{ik}(\mathbf{x})$. The location of each nanocrystal $\mathbf{X}_k$ is estimated as

$$\mathbf{X}_k = \frac{1}{N_k} \sum_i \mathbf{x}_{ik}, \tag{4}$$

with N_k the number of emitters in crystal k. To add the PDFs of molecules in different NCs, we find the most likely orientation of molecules Φ_k in crystal k, and transform the PDFs with a translation and a rotation, such that

$$\hat{G}_{ik}(\mathbf{x}) = R_{O,-\Phi_k}[G_{ik}(\mathbf{x} + \mathbf{X}_k)] \tag{5}$$

where $R_{O,-\Phi_k}$ denotes a clockwise rotation through an angle Φ_k about the origin $\mathbf{x} = (0, 0)$. This transformation is shown in Figure S2d-f for the three examples of Figure S2a-c. For the average shape of the printed NCs in Figure 5e, we calculated the sum of transformed PDFs, i.e. $\sum_{i,k} \hat{G}_{ik}(\mathbf{x})$.

Figure S3 presents a histogram of the measured distance between emitters $\|\mathbf{x}_{ik} - \mathbf{x}_{jk}\|$. The maximum distance found between two pairs in one NC is 846 nm. The localization accuracy in our extended measurements is limited to approximately $\sigma = 20$ nm. As a result, the maximum size of the printed NCs is below 850 nm.

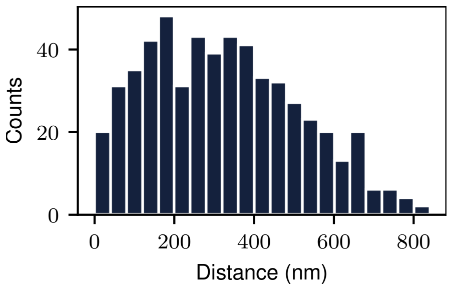

FIG. S3. Distribution of distances between 560 pairs of molecules.

REFERENCES

[1] Fleury, L.; Zumbusch, A.; Orrit, M.; Brown, R.; Bernard, J. Spectral diffusion and individual two-level systems probed by fluorescence of single terrylene molecules in a polyethylene matrix. *J. Lumin.* **1993**, *56*, 15–28.

[2] Barkai, E.; Silbey, R.; Zumofen, G. Lévy Distribution of Single Molecule Line Shape Cumulants in Glasses. *Phys. Rev. Lett.* **2000**, *84*, 5339–5342.
